# Intermediate uveitis

**DOI:** 10.4103/0301-4738.58469

**Published:** 2010

**Authors:** B Manohar Babu, S R Rathinam

**Affiliations:** Uvea Clinic, Aravind Eye Hospital, Coimbatore, India; 1 Aravind Eye Hospital, Madurai, India

**Keywords:** Intermediate uveitis, pars planitis

## Abstract

Intermediate uveitis (IU) is described as inflammation in the anterior vitreous, ciliary body and the peripheral retina. In the Standardization of Uveitis Nomenclature (SUN) working group's international workshop for reporting clinical data the consensus reached was that the term IU should be used for that subset of uveitis where the vitreous is the major site of the inflammation and if there is an associated infection (for example, Lyme disease) or systemic disease (for example, sarcoidosis). The diagnostic term pars planitis should be used only for that subset of IU where there is snow bank or snowball formation occurring in the absence of an associated infection or systemic disease (that is, “idiopathic”). This article discusses the clinical features, etiology, pathogenesis, investigations and treatment of IU.

Uveitis or intraocular inflammation has many subtypes and many potential associations with systemic conditions and has always been one of the most challenging diagnoses in ophthalmology. Classification of uveitis into subtypes helps immensely in diagnosis, treatment and prognosis of a patient's condition. This article outlines epidemiology, clinical features, complications, etiopathogenesis, pathology, differential diagnosis, investigations and treatment. A Medline search was conducted for relevant articles published in English. Articles were analyzed for content and evidence level.

Intermediate uveitis (IU), pars planitis, chronic cyclitis, peripheral uveitis, vitritis, cyclochorioretinitis, chronic posterior cyclitis and peripheral uveoretinitis are the nam es that have been used to describe inflammation in the anterior vitreous, ciliary body and the peripheral retina.[1]

The IUSG (International Uveitis Study Group) suggested the term IU to denote an idiopathic inflammatory syndrome, mainly involving the anterior vitreous, peripheral retina and the ciliary body with minimal or no anterior segment or chorioretinal signs.[2]

More recently during the Standardization of Uveitis Nomenclature (SUN) working group's international workshop for reporting clinical data the consensus reached was that the term IU should be used for that subset of uveitis where the vitreous is the major site of the inflammation, and that the presence of peripheral vascular sheathing and macular edema should not change the classification. The diagnostic term pars planitis should be used only for that subset of *IU* where there is snow bank or snowball formation occurring in the absence of an associated infection or systemic disease (that is, “idiopathic”). If there is an associated infection (for example, Lyme disease) or systemic disease (for example, sarcoidosis), then the term IU should be used.[3]

## Epidemiology

In the Western literature IU has been reported in 1.4-22% of uveitis patients.[1,4–7] In India the percentage of IU varies from 9.5-17.4%.[8–10] The prevalence is estimated to be 5.9/100,000 and incidence 1.4/100,000.[11] In a South India-based study the prevalence of active IU was 0.25%.[12] Though the disease affects patients in all age groups, it is predominantly seen in the third and fourth decade.[5,10,13] Bilaterality is seen in 70-90% in the Western literature[1,13] and is 37.6% in a South India-based study.[10] No definite gender predilection is seen.

IU is not hereditary though it has been observed in families. Human leucocyte antigen (HLA) studies have shown common HLA haplotypes in a few families.[14–18] It has been shown that patients who are HLA-DR15-positive and have IU may have systemic findings of another HLA-DR15-related disorder- multiple sclerosis, optic neuritis, and narcolepsy.[19]

IU accounts for 10-12% of all uveitis seen in children.[20]

## Clinical Features

Patients with IU present with minimal symptoms, floaters or blurred vision. In severe cases they can present with visual loss due to aggregation of floaters in the vitreous or due to macular edema. The anterior chamber may be quiet or may have signs of inflammation in the form of keratic precipitates (KP's) or flare and cells, which are usually minimal. Posterior synechiae may or may not be seen, if present, are seen usually involving the inferior iris. Vitritis is a characteristic feature of IU, and it is typically described as vitreous haze ranging from trace to 4+.[3]

**Figure 1:**
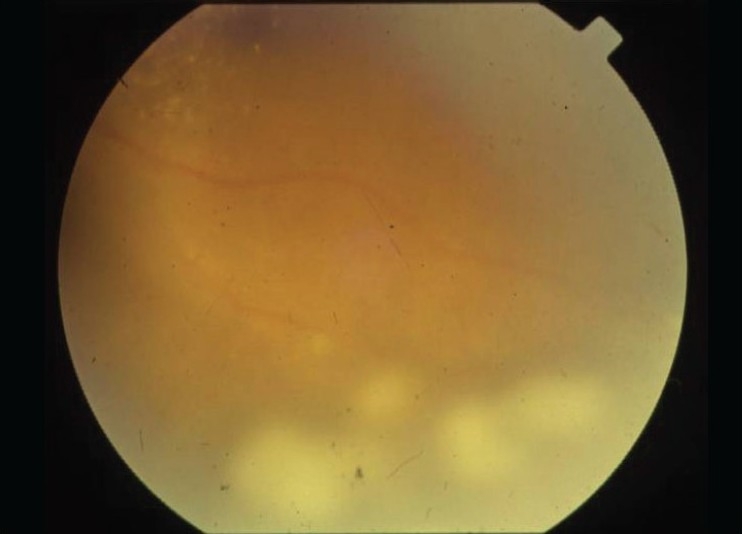
Pars plana snowball exudates

Vitreous snowballs [Fig. 1] typically are yellow-white inflammatory aggregates, and are found in the midvitreous and inferior periphery. Snowbanks are exudates on the pars plana, when present are usually found inferiorly, but may also extend 360 degrees of the retinal periphery. Snowbanking is usually associated with the more severe form of the disease, and warrants aggressive therapy. Retinal changes in IU include tortuosity in arterioles and venules, sheathing of peripheral veins, neovascularizations and retinal detachments.[21–23]

## Complications

IU is most often a benign form of uveitis. Its complications are due to its chronicity, and if left untreated can lead to blindness. The incidence of glaucoma in acute uveitis is 7.6% and in patients with chronic uveitis the incidence of glaucoma at one and five years is 6.5% and 11.1% respectively. There was no statistically significant difference between anterior, intermediate, posterior and panuveitis entities and the presence of glaucoma was associated with an increased risk of visual loss.[24] Active inflammation, steroid usage, increasing age, and number of years since diagnosis are significantly correlated with raised intraocular pressure (IOP).[25]

Cataracts occur in 15-50% of eyes. Typically they are located posterior or anterior subcapsularly, or both, or posterior cortically. Posterior polar cataracts have been reported as well. The incidence of cataracts increases with the duration and severity of the disease. If treated earlier with immunosuppressives rather than corticosteroids cataract formation is less severe.[1,23,26]

**Figure 2:**
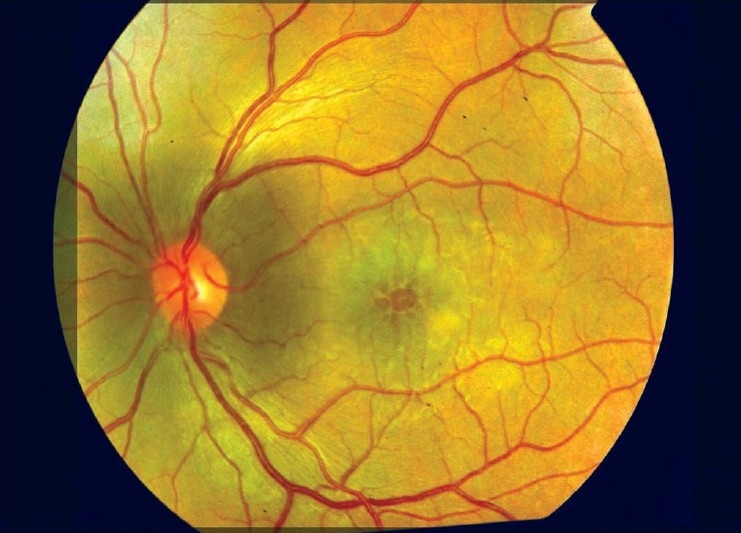
Cystoid macular edema

Macular edema and maculopathy are the most common causes of visual loss [Fig. 2]. Incidence varies from 12 to 51%. Like cataract their incidence increases with the duration and severity of the disease.[1,23,26] Epiretinal membranes occurred in 34.6-36% eyes, which was not related to duration of disease or chronic cystoid macular edema (CME).[27,28]

Retinal vasculitis in the form of periphlebitis is found in 16-36%.[27–29] It may induce neovascularization and cyclitic membrane formation.[1] Retinal detachments (RD) occurred in 2.2-51% eyes.[23,26–28,30] Exudative RD has been seen secondary to inflammation in IU.[1] But the most common forms to be seen are vitreous traction secondary to longstanding vitreous inflammation and subsequent peripheral hole formation.[26]

Peripheral neovascularization with and without vitreous hemorrhage was seen in 6.5% by Malinowski *et al*.[27] Optic nerve involvement in the form of disc edema is seen in 3-38.6% of eyes with IU.[23,29,31] Optic neuritis with or without multiple sclerosis was seen in 7.4% of eyes with pars planitis.[27]

## Etiopathogenesis

IU may be initiated by an unknown antigen, leading to a clinical picture of vasculitis and vitreous cells. It is possible that the antigen may be infectious because IU is seen in infectious diseases like Lyme's, syphilis and cat-scratch fever. The disease may be autoimmune – as IU is also seen in non-infectious disorders like multiple sclerosis and sarcoidosis.[1] Type II collagen in the vitreous may be an autoantigen in some patients.[32]

IU seems to be a T-cell-mediated disease, as it can be reproduced in experimental models using retinal S antigen/ interphotoreceptor retinoid binding protein (IRBP)/ hyaluronic acid, and the disorder responds well to immunosuppression. Lymphocytic infiltration of the retinal venules leads to the clinical picture of vasculitis. Major histocompatibility complex (MHC) Class II antigen expression was found on the vascular endothelium, which could be a part of the initiating process in the recruitment of activated T-cells to stimulate a local vasculitis, leading to vitreous inflammation.[1]

T-cells are the predominant cell type in the vitreous in IU- up to 95% of all cells, of which CD4+ cells are 35-90%.[33,34] Macrophages are the second most important cells seen. In active inflammation epitheloid cells and multinucleated giant cells are seen.[35] A 36 kDa protein (p-36) is found in elevated concentrations in the blood of many patients with active pars planitis. The levels of this protein correlated with disease activity. Its role in the etiopathogenesis of pars planitis is unknown.[36] HLA associations include HLA-DR, B8, and B51, the most significant being HLA-DR which occurs in 67-72% of patients. In a small study of 18 patients with IU, 72% were HLA-DR 15 positive and of these 32% had IU.[19,37] HLA association identifies individuals at risk and is not a diagnostic marker.

## Pathology

Histological studies of the peripheral retina and ciliary body demonstrate condensed vitreous, fibroblasts, spindle cells, lymphocytes and blood vessels and prominent lymphocyte cuffing of retinal veins.[37] Pars plana exudates appear to consist of loose fibrovascular layer containing scattered mononuclear inflammatory cells and a few fibrocyte-like cells adjacent to the hyperplastic nonpigmented epithelium of the pars plana. This fibroglial tissue consists of vitreous collagen, Muller cells and probable fibrous astrocytes.[38]

## Differential Diagnosis

IU has been associated with infectious conditions like Lyme's disease (*Borrelia burgdorferi*), toxoplasmosis, toxocariasis, tuberculosis, syphilis, human lymphotropic virus Type 1 (HTLV-1), Epstein-Barr virus and cat-scratch disease (*Bartonella henselae, B quintana*). It is also associated with noninfectious entities like multiple sclerosis, sarcoidosis and intraocular lymphoma.

Multiple sclerosis: About 3-27% of patients with multiple sclerosis (MS) develop IU/pars planitis,[39,40] and 7.8-14.8% of patients with IU/pars planitis develop MS.[27,41] IU characterized by pars plana snowbanks, retinal periphlebitis (in 5-20%) and panuveitis are the commonest manifestations of MS and up to 95% are bilateral.

Sarcoidosis: About 23-26% of patients with sarcoidosis develop IU,[42,43] and 2-10% of patients with IU develop sarcoid disease.[41,44] The typical ocular findings, CME, optic disc swelling, periphlebitis, and retrobulbar optic neuritis were seen in patients with IU, both with or without sarcoidosis.[44] It is commonly bilateral, and presents as IU and granulomatous anterior uveitis.[45]

Intraocular lymphoma: Two-thirds of intraocular lymphomas are a manifestation of a primary central nervous system lymphoma (PCNSL) arising outside the lymphatic system and are localized in the brain, the meninges or the spinal cord. In 10-20% the disease commences as vitreous or retinal infiltrates mimicking uveitis and 95% of PCNSL are non-Hodgkins B-cell lymphomas. Mean age at presentation was 63.5 years with a female to male ratio of 6 to 4.[46] The diagnostic procedures are vitreous biopsy, neurologic history, cerebrospinal fluid (CSF) studies, brain magnetic resonance imaging (MRI).

Syphilis: In the eye, uveitis is the commonest presentation of syphilis. In a case series of Fluorescent Treponemal Antibody Absorption (FTA-ABS)-positive syphilis patients with uveitis, though anterior uveitis, nongranulomatous (in 62%), was the commonest presentation seen, IU was observed in 10.3%.[47] Anterior uveitis, both granulomatous and nongranulomatous, posterior uveitis, panuveitis, vitritis, vasculitis, retinitis, placoid choroiretinitis and optic nerve involvement are also seen in eyes with syphilitic uveitis. History, systemic and ocular examination and serologic testing with venereal disease research laboratory (VDRL) and FTA-ABS tests, should exclude the diagnosis of syphilis.[1,47] IU has been described to occur in Lyme's disease caused by another spirocheate- *Borrelia burgdorferi*, both in adults and in children.[48,49]

Tuberculosis: Infection with *Mycobacterium Tuberculosis* can induce a similar picture of IU. A thorough history, systemic and ocular examination, chest X-ray, and skin testing are necessary. Finding of granulomatous iris nodules and/or choroidal granulomas should alert us to suspect tubercular etiology.[1]

Others: IU has been reported in children with renal diseases, tubulointerstitial nephritis and uveitis syndrome (TINU syndrome), and mesangial glomerulonephritis.[50,51] IU has also been reported to occur in antineutrophil cytoplasmic antibody (ANCA)-associated vasculitis,[52] and in post-streptococcal uveitis.[53] A case of autoimmune lymphoproliferative syndrome (ALPS) presenting with bilateral uveitis was seen and control of the IU required sustained doses of topical and periocular corticosteroids as well as systemic cyclosporine.[54] IU is also a rare manifestation of Behçet's disease and AIDS, and chronic propionibacterial endophthalmitis.[55] Uveitis occurs in 14.5% of patients with (HTLV-I) disease, and manifests as IU in 78.6%.[56,57]

Uveitic entities like peripheral toxoplasmosis, toxocariasis, endogenous endophthalmitis, acute retinal necrosis (ARN), retinal vasculitis associated with Eale's disease, Fuch's heterochromic cyclitis with vitreous haze and Vogt-Koyanagi-Harada disease with vitritis and retinal detachments must be ruled out before starting therapy.

## Diagnosis

The diagnosis of IU is based on clinical findings. Patient's complaints of defective vision and/or floaters in the absence of pain, redness, photophobia should alert the ophthalmologist. Presence of vitreous cells that outnumber anterior chamber cell infiltration, vitreous snowballs, and the presence of pars plana exudation, suggest IU. Laboratory and ancillary tests are not necessary to establish the diagnosis; however, with a careful history, ocular and systemic examination together with laboratory studies, we may be able to exclude an associated disorder.

The patient's history should concentrate on the duration of symptoms, the number of recurrences, and findings that might be associated with systemic disorders. Fever, fatigue, or night sweats are typical signs of sarcoidosis and tuberculosis, whereas loss of sensitivity or paresthesias of the hands, arms, or legs are suggestive of possible MS. Signs of dermatitis may point to Lyme disease, tuberculosis, or syphilis, whereas arthritis of the knee may suggest the possibility of Lyme's, disease, and contact with cats may raise the possibility of Bartonella infection.[1]

A routine baseline workup comprising complete blood count which includes total and differential count/ hemoglobin/ platelet count, erythrocyte sedimentation rate, purified protein derivative skin test (PPD) and chest X-ray are mandatory. Total count is increased in –infections, chronic inflammation and autoimmune disease. Infections predominantly cause an increase in neutrophils, and lymphocytosis may indicate a possible tuberculosis etiology. Chest X-ray studies may disclose findings indicative of sarcoidosis or tuberculosis.

A PPD test is needed to exclude tuberculosis/sarcoidosis.

In cases of IU, only a few laboratory and serologic tests are necessary. These tests include determination of the angiotensin-converting enzyme (ACE) level. Serologic testing for cat-scratch disease, syphilis, and Lyme's: disease should be seriously considered in cases of IU.

Subclinical pulmonary sarcoidosis, undetectable by chest X-ray study, may be detected via computed tomography (CT) of the chest or by gallium scan, or both. A combination of serum ACE level and whole-body gallium scan increases the diagnostic specificity without affecting sensitivity in patients with clinically suspicious ocular sarcoidosis who have normal or equivocal chest radiographs. Fluorescein angiography (FA) alerts one to the presence of vasculitis, areas of retinal nonperfusion and neovascularization and CME. It is useful in following up a patient as well.

Using ultrasound biomicroscopy (UBM), it is possible to demonstrate pars plana exudates, and even inflammatory cell aggregates in the vitreous. Ultrasonography (USG) can be done to rule out RD, intraocular tumors.

Diagnostic vitrectomy is done in cases when tumors are suspected, in patients with severe vitreous inflammation where retinitis, endophthalmitis cannot definitely be excluded and in cases where response to medical therapy is refractory.[1]

**Intermediate uveitis in children:** Uveitic entities with an etiologic diagnosis seen in the pediatric age group are: juvenile idiopathic arthritis (30%), toxoplasmosis (3.3%), HLA-B27-associated iritis (1.89%), acute retinal necrosis (1.1%), tubulointerstitial nephritis associated anterior uveitis (1.1%), Kawasaki-related anterior uveitis (0.7%), and Vogt–Koyanagi–Harada syndrome (1.1%), isolated cases of sarcoid uveitis, multifocal choroiditis and panuveitis, Behçet's disease, *Herpes simplex virus* keratouveitis, masquerade syndrome, late-onset retinoblastoma, systemic lupus erythematosus, sympathetic ophthalmia, toxocariasis, V*aricella-zoster virus* iritis, and infectious endophthalmitis.

IU accounts for 1.8-29% of uveitis seen in the pediatric age group. All cases were reported to be idiopathic IU in three studies reported from various parts of the world.[58–61] This is in contrast to a report from south India. Idiopathic uveitis in children accounted for only 25.5% of cases, whereas infectious uveitis was found in 58%.[10] Mean age of onset of uveitis is 8.5-10.9 years. There is a male preponderance. Bilateral involvement is seen in 84-94% patients. Chronicity of uveitis is 84-100%. Mean time to remission is 6.4 years. Common complications seen are: disc edema, CME, cataract, glaucoma, and band-shaped keratopathy. Epiretinal and neovascular membranes occur. Snowbanking is seen in 28% of patients.[58,62]

Since the etiology of IU remains elusive in most cases, the therapy is mainly symptomatic. The presence of CME is an indication for treatment with periocular corticosteroid injections with or without a short-term course of systemic corticosteroids. Boer *et al*. conclude that IU in children might resolve after several years and, despite a high ocular complication rate, severe visual loss is uncommon.[62]

## Treatment

Treatment is directed at the cause, if detected. Malignancies need to be ruled out. Treatment discussed here is nonspecific anti-inflammatory therapy for IU. Indications for treatment are decrease in visual acuity to <20/40 due to macular edema, vitreous haze[63] and retinal vasculitis.

### Drug therapy

Kaplan first advocated a four-step treatment of IU in 1984.[64] A discussion of various treatment modalities follows: Corticosteroids: Corticosteroids are indicated when the visual acuity drops due to vitritis, CME or progression of neovascularization at the vitreous base. Periocular corticosteroids are the first line of management. Local injection of depot preparation of either a long-acting methylprednisolone (40 mg) or triamcinolone acetonide (20 mg) is given either through the posterior sub-tenon route or retroseptally through the lower lid, and can be spaced —two to four weeks apart. Complications of periocular injections are increased IOP, cataract, aponeurotic ptosis and allergic reactions with conjunctival breakdown. Repeated injections may cause enophthalmos and orbital scarring. Improvement in at least two Snellen lines was seen in 12/18 patients at a median of three weeks.[65]

If local therapy is not effective or bilateral severe disease is seen at presentation oral corticosteroids are indicated. Oral prednisolone is started at 1 mg/kg/day with gradual tapering after two weeks and guided thereafter by the clinical response. Ideally, the disease should be controlled with 5 mg or less daily. Eyes treated with oral and periocular steroids improved vision-wise[66] and angiography-wise.[23]

Intravitreal triamcinolone (IVTA) may be an alternative to periocular injections in refractory cases though they carry the risk of RD, vitreous hemorrhage, IOP elevation and endophthalmitis. IVTA was associated with an improvement in vision of more than two lines in 50% of the eyes within 12 weeks after injection, as reported by Hogewind *et al*., where 33 eyes were treated with IVTA for uveitic CME that was refractory to topical steroids, oral prednisone, or a combination. Cataract and glaucoma were the common side-effects.[67]

Immunomodulatory therapy may be considered at this point if corticosteroids fail. Methotrexate, azathioprin, cyclosporine, mycophenolate mofetil, tacrolimus have been used in treating IU. Cyclophosphamide and chlorambucil have been used in refractory uveitis. Newer biologic agents are being used as well.

Antimetabolites/antiproliferative drugs: Methotrexate (MTX), a folate analog which inhibits dihydrofolate reductase, is used at a dose of 7.5-25 mg per week oral/subcutaneous. Though its potential side-effects are gastro intestinal (GI) upset, fatigue, hepatotoxicity and pneumonitis, it is effective and safe for chronic anterior and IU in children.[68]

Azathioprine, a purine nucleoside analog, alters purine metabolism. It is used at a dose of 50-150 mg per day in divided doses orally. Its potential side-effects are GI upset and hepatotoxicity.

Mycophenolate mofetil (MMF) acts by inhibiting purine synthesis, prevents replication of T and B lymphocytes by selectively inhibiting inosine-5-monophosphate dehydrogenase. It is used at a dose of 1-3 mg per day in divided doses orally. Diarrhea, nausea, and GI ulceration are its potential side-effects. The rate of MMF discontinuation because of side-effects was low, GI disturbance was the commonest side-effect seen.[69] It has been found to be safe in children when used alongside oral corticosteroid.[70] Galor *et al*. compared all the three antimetabolites in a cohort of patients with ocular inflammation which included patients with IU in all three groups, and concluded that time to control of ocular inflammation is faster with mycophenolate than with methotrexate.[71]

### Inhibitors of T-cell signaling

Cyclosporine (CsA): Inhibits NF-AT (nuclear factor of activated T-cells) activation, and is used at a dosage of 2.5-5.0 mg per kg per day in divided doses orally. Known toxic effects are nephrotoxicity, hypertension, gingival hyperplasia, GI upset and paresthesias. At the National Institute of Health (NIH) cyclosporine is the first-line steroid-sparing agent in IU.[72]

Tacrolimus: Inhibits NF-AT activation, and is used at a dose of 0.1-0.2 mg per kg per day orally. Nephrotoxicity, hypertension and diabetes mellitus are its known potential complications. Tacrolimus's efficacy for the treatment of uveitis is maintained long-term, and its cardiovascular risk profile is excellent.[73]

Biologic response modifiers: Newer anti-inflammatory drugs like daclizumab, infliximab, eternercept, interferon alpha are being increasingly used as first-line, second-line drugs in the management of refractory uveitis.

Daclizumab: humanized monoclonal anti-IL-2 receptor alpha antibody. It binds to the alpha subunit of IL-2 receptor thereby suppressing autoreactive T-cells. It is used at a dose of 1.0 mg per kg IV every two weeks for five doses. High-dose daclizumab can reduce inflammation in active uveitis and is well tolerated but there may be a potential increased risk of infection associated with immunosuppression.[74]

### Surgical Therapy

Cryotherapy and laser photocoagulation: If drug therapy has failed or recurrent inflammation is seen despite corticosteroid use, cryotherapy or laser photocoagulation may be used to control the disease.[1] Cryotherapy before immunomodulatory therapy is also the preferred practice as described in Western literature.[75] Peripheral ablation of the pars plana snowbank with cryotherapy or indirect laser photocoagulation to the peripheral retina can be done. Cryotherapy is performed by applying a double row, single freeze of transconjunctival cryopexy to the pars plana and posterior to it, extending to an area 1 o'clock-h beyond all evidence of disease activity. Photocoagulation burns may be placed confluently in three or four rows just posterior to the snowbank. The rationale for these procedures is to treat the neovascularization associated with pars plana exudation and vitritis and to destroy the vascular component of the peripheral retinitis or vitritis, thus eliminating the entrance site for inflammatory mediators into the eye.[1,72] Cryotherapy decreases vitritis and improves visual acuity.[76] It has also been shown to decrease fluorescein leakage in these areas. Laser photocoagulation seems to be as effective as cryotherapy in treating inflammation and peripheral neovascularization.[68] Cryotherapy is not universally used due to reported increased incidence of post-treatment RD. It can, however, be considered in patients who have neovascularization of vitreous base and a history of vitreous hemorrhage.

Vitrectomy: Decreased inflammatory disease has been reported after pars plans vitrectomy (PPV) for chronic inflammation in patients with IU. PPV is an important means of correcting structural complications of uveitis, helps in decreasing inflammation in the anterior chamber and in the vitreous and in reduction of anti-inflammatory medication postoperatively. It helps in improving visual outcome and is beneficial in reducing CME.[77]

Cataract: Cataracts are a frequent complication that result from both, chronic inflammation and corticosteroid therapy. Phacoemusification and intraocular lens (IOL) implantation is safe in IU/pars planitis.[78] Visual acuity of 20/40 or better was seen in 88% of patients following cataract surgery and IOL implantation in whom control of inflammation for three months preoperatively was achieved.[79] Control of inflammation can be achieved with use of corticosteroids- topical, periocular, oral with or without immunosuppressive therapy.[80]

Our suggested algorithm for treatment of IU is as follows:

Step 1: Periocular steroids administered by local injection of depot corticosteroids may be repeated every four weeks until three to four injections have been administered. Generally, the inflammation responds and the CME improves. IVTA may be an alternative to periocular injections in refractory cases.

Step 2: If local therapy is not effective or bilateral severe disease is seen at presentation oral corticosteroids are indicated.

Step 3: Systemic immunomodulatory therapy is indicated in the treatment of bilateral disease, and can be considered if corticosteroids fail, are not tolerated or are contraindicated.

Step 4: If corticosteroids fail, or if corticosteroids and immunomodulatory therapy are contraindicated, and if pars plana snowbanks are present, peripheral ablation with cryotherapy or indirect laser photocoagulation to the peripheral retina can be done.

Step 5: If all the treatment modalities fail to control inflammation, PPV with induction of posterior hyaloidal separation and peripheral laser photocoagulation to pars plana snowbank may be performed, along with immunomodulatory therapy.
